# Prokaryotic diversity and community structure in the rhizosphere of Lantana weed (*Lantana camara* L.)

**DOI:** 10.3389/fpls.2023.1174859

**Published:** 2023-04-21

**Authors:** Upasana Gola, Shilippreet Kour, Tanvir Kaur, Kahkashan Perveen, Najat A. Bukhari, Jamilah A. Alsulami, Damini Maithani, Hemant Dasila, Manali Singh, Deep Chandra Suyal

**Affiliations:** ^1^Department of Microbiology, Akal College of Basic Sciences, Eternal University, Baru Sahib, India; ^2^Department of Genetics, Plant Breeding and Biotechnology, Dr. Khem Singh Gill Akal College of Agriculture, Eternal University, Baru Sahib, India; ^3^Department of Botany & Microbiology, College of Science, King Saud University, Riyadh, Saudi Arabia; ^4^Microbiology Department, Howard University, Washington, DC, United States; ^5^School of Biotechnology, IFTM University, Moradabad, India; ^6^Department of Biotechnology, Invertis University, Bareilly- Lucknow National Highway, Bareilly, India; ^7^Vidyadayini Institute of Science, Management and Technology, Sajjan Singh Nagar, Bhopal, Madhya Pradesh, India

**Keywords:** Illumina HiseqX, PICRUSt analysis, bacterial diversity, metagenomics, weed plants

## Abstract

Lantana weed (*Lantana camara* L.) is among the most noxious weeds in the world. Keeping in mind its invasive behavior and great ecological tolerance, it becomes imperative to analyze the structure and function of associated microbiome. In this perspective, Illumina-based metagenome sequencing was performed to gain a better understanding of prokaryotic diversity and community structure in the rhizosphere soil of *L. camara* L. The organic carbon, nitrogen, phosphorus, and potassium contents in the rhizosphere soil were 0.91% (± 0.21%); 280 Kg ha^-1^ (± 4.02 Kg ha^-1^), 54.5 Kg ha^-1^ (± 3.12 Kg ha^-1^), and 189 Kg ha^-1^ (± 6.11 Kg ha^-1^), respectively. The metagenome analysis revealed the existence of 41 bacterial and 2 archaeal phyla, with only 12 showing ≥1% abundances. Pseudomonadota was the dominant phylum with 31.3% abundance, followed by Actinomycetota (20.9%). Further, 54 different genera were identified with the highest abundance of *Devosia* (2.8%). The PICRUSt analysis predicted various functional traits in the soil metagenome, with general cellular functions dominating, followed by stress tolerance. Moreover, 10% of the functions were associated with nitrogen fixation, phosphate solubilization, and potassium mobilization. In conclusion, the present study revealed the existence of diverse prokaryotic communities in the rhizosphere of the *L. camara* L. which was primarily associated with stress response and plant growth promotion. To the best of our knowledge, this study documents for the first time the *L. camara* L. microbiome. Furthermore, the identified genera can be explored for agricultural needs in future.

## 1 Introduction

Weeds are undesirable plants that can flourish in diverse climates, habitats, and soil conditions without any input or care. *L. camara* L. is among the most noxious weeds in the world. It is native to tropical America and was introduced into many other countries as a hedge and ornamental plant (Sharma et al., 2005). The diverse and widespread geographic distribution reflects its ability to withstand stress. This ecological tolerance depends strongly on its allelochemicals and rhizosphere microorganisms (Trognitz et al., 2016; Sahu et al., 2021). It can be supported by the fact that plant associated microorganisms influence host characteristics, *viz.* disease resistance, nutrient acquisition, flowering time, biomass production, and abiotic stress tolerance (Jones et al., 2009; van Overbeek et al., 2011; Marasco et al., 2012). Furthermore, beneficial rhizosphere bacteria have been reported to alter gene expression patterns in several agriculturally important plant species and also enhance plant productivity under abiotic stress conditions (Marasco et al., 2012; Panke-Buisse et al., 2015). Similarly, van Overbeek et al. (2011); Bahulikar et al. (2014) and Luu et al. (2021) have identified drought tolerating bacteria from the rhizosphere of weed plants. In another study, Sturz et al. (2001) have reported higher abundance of plant growth-promoting bacteria (PGPB) in a weed rhizosphere in comparison to the crop plants of the same agricultural field. In short, the weed plants harbour a hidden treasure of beneficial microorganisms that can be explored for various agricultural purposes. Assessment of microbial diversity and community structure are the crucial and foremost steps in utilizing the full potential of given microbiome. They give an idea about microbial gene pool, their distribution pattern and functional attributes in a particular niche. Moreover, such studies are proven very useful in making the isolation strategies for specific microbial genera because major fraction of the diversity is still uncultivable in the lab (Kumar et al., 2019). In case of *L. camara* L. very scarce information is available about the associated microorganisms. Therefore, our study provides an insight to the prokaryotic diversity and community structure available in its rhizosphere. To the best of our knowledge, this is the first next generation sequencing (NGS) based diversity analysis of the *L. camara* L. microbiome. The genera identified in this study can be targeted to be developed as bioinoculants for native crops of Himachal Pradesh. In this perspective, these efforts are a step forward to utilize and bio-prospect the indigenous microbial diversity for agriculture sustainability in future.

## 2 Materials and methods

### 2.1 Sampling sites and sample collection

Soil samples were collected during October 2021 from the rhizosphere of the *L. camara* L. plant of the Baru Sahib region, situated at 30.7537° N, 77.2965° E, in the Sirmaur district of Himachal Pradesh, India. This region has average annual temperature, rainfall and humidity is of 22°C, 70% and 159 mm, respectively. The soil samples were collected from the rhizosphere of three *L. camara* L. plants located at least at 100 m distance by using a random sampling technique. The upper surface of the soils was removed to avoid any contamination. Soil samples from the vicinity of the roots and a depth upto 15 cm were collected. The samples were collected with the help of sterile spatula in a sterilized sampling bag and immediately transferred at 4°C till further use. All three soil samples were pooled later to make a single composite sample for metagenome sequencing.

### 2.2 Soil analysis

Oxidizable organic carbon of rhizosphere soils was measured by using the method given by Walkley and Black (1934). Further, Alkaline permanganate procedure estimated the available nitrogen content (Subbiah and Asija, 1956). Available phosphorous content was determined through (Olsen, 1954). Similarly, available potassium content was estimated *via* Jackson method (Jackson, 1973). Further, colony-forming units (CFU) of total bacteria, nitrogen fixers, and P-solubilizers were calculated by using the serial dilution plating technique on Nutrient Agar, Burk’s medium, and Pikovskaya’s agar, respectively. All the methodologies and procedures are provided as **Supplementary Material** in detail.

### 2.3 DNA extraction

The soil DNA was extracted through Power Soil™ DNA isolation kit (Mobio Lab. Inc., USA). Extracted DNA was further screened qualitatively and quantitatively *via* agarose gel electrophoresis and spectrophotometry techniques, respectively.

### 2.4 Metagenome sequencing and bioinformatics analysis

The prokaryotic diversity of the *L. camara* L. rhizosphere was determined by sequencing V3-V4 amplicons through the Illumina HiseqX sequencing machine. The analysis was performed in triplicate. Library size was determined by Agilent Technologies 2100 Bioanalyzer instrument using a DNA 1000 chip. Furthermore, Library quantity was analyzed through Illumina qPCR quantification and rapid library standard quantification procedures. FastQC (v0.11.7) software (Andrews, 2010) was used to check the quality of raw reads and then clean amplicons were further processed through TrimGalore (v0.5.0) software (Krueger, 2012). The QIIME software package (v. 2.0) was used for removing singletons, and assigning operational taxonomy units (OTU) to the remaining sequences (Bolyen et al., 2019; Kumar et al., 2019; Suyal et al., 2021). PICRUSt software (v. 2.0) (Langille et al., 2013) was performed to identify the functional traits of the microbiome. The 97% similarity threshold was used during OTU-picking.

The NGS data generated in this study have been deposited to the NCBI database under the accession number SAMN29618131 and Bio-project ID PRJNA85716.

### 2.5 Statistical analysis

All the analyses were performed in triplicates. The data are represented as the mean ± standard error.

## 3 Results

Soil analysis revealed the physiochemical properties of the *L. camara* L. rhizosphere soil. It had pH of 6.4 ( ± 0.2) with an organic carbon content of 0.91% (± 0.21). Further, nitrogen (N), phosphorus (P), and potassium (K) contents of the soil were 280 Kg ha^-1^ (± 4.02), 54.5 Kg ha^-1^ (± 3.12), 189 Kg ha^-1^ (± 6.11), respectively. The good amount of nitrogen and phosphorus content in rhizosphere soil is well related with the abundance of N-fixing and P-solubilizing bacteria in the soil (**Table 1**). N-fixing bacteria fix atmospheric nitrogen as nitrate while P-solubilizing bacteria liberate phosphate ions in the soil and thus make it nutrient rich.

**Table 1:** Physicochemical properties and viable cell counts in the rhizosphere of *L. camara* L. Each value is the mean of three replicates.

Soil texture	Silty clay
Soil colour	Yellowish-brown
pH	6.4 (± 0.2)
Organic carbon (%)	0.91 (± 0.21)
Available N (Kg ha^-1^)	280 (± 4.02)
Available P (Kg ha^-1^)	54.5 (± 3.12)
Available K (kg ha^-1^)	189 (± 6.11)
Total viable bacterial count (CFU g^-1^ of soil)	2.3 x10^6^ (± 1.7 x10^6^)
Nitrogen fixers (CFU g^-1^ of soil)	3.35 x10^5^ (± 6.5 x10^4^)
P-solubilizers (CFU g^-1^ of soil)	7.5 x10^4^ (± 4.5 x10^3^)

Values in bracket indicate standard error.

### 3.1 Soil metagenome sequencing

A total number of 394,273 raw reads were obtained from the rhizosphere soil metagenome with a sequence length of 251 bp and 57% GC content. After quality checking and clustering of homologous sequences, a total of 30,024 OTUs were identified.

### 3.2 Taxonomic distribution of the OTUs

**Figure 1:**
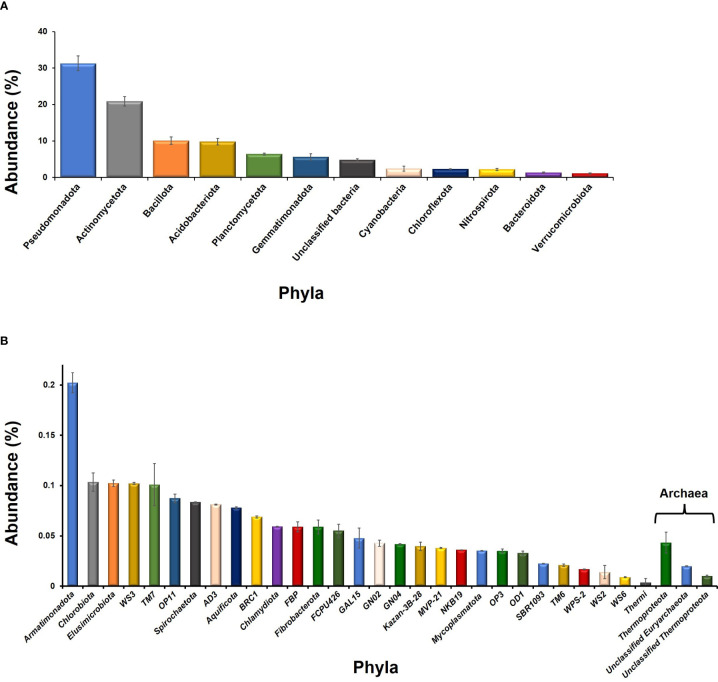
Taxonomic distribution of the phyla observed in the rhizosphere of *L. camara* L. Whereas, phyla having ≥1% abundance and ≤1% abundance are plotted as **(A, B)**.

The 99.7% OTUs were from domain bacteria while 0.7% belonged to domain archaea. A total of 41 bacterial phyla were observed among which only 12 have shown ≥1% abundance (**Figure 1A**). Taxonomic distribution of the bacterial OTUs showed that Pseudomonadota was the dominant phylum (31.3%) followed by Actinomycetota (20.9%), Bacillota (10.1%), and Acidobacteriota (9.8%), and Planctomycetota (6.4%). Furthermore, Cyanobacteria and Bacteroidota had 2.4 and 1.3% abundance, respectively. A major fraction of the OTUs (4.8%) belonged to unclassified bacterial members (**Figure 1A**). Among archaea, most of the OTUs were from unclassified members of Euryarchaeota and Thermoproteota (**Figure 1B**). Few of the OTUs also belonged to “yet not cultivated” taxa i.e., Candidatus Nitrososphaera.

**Figure 2:**
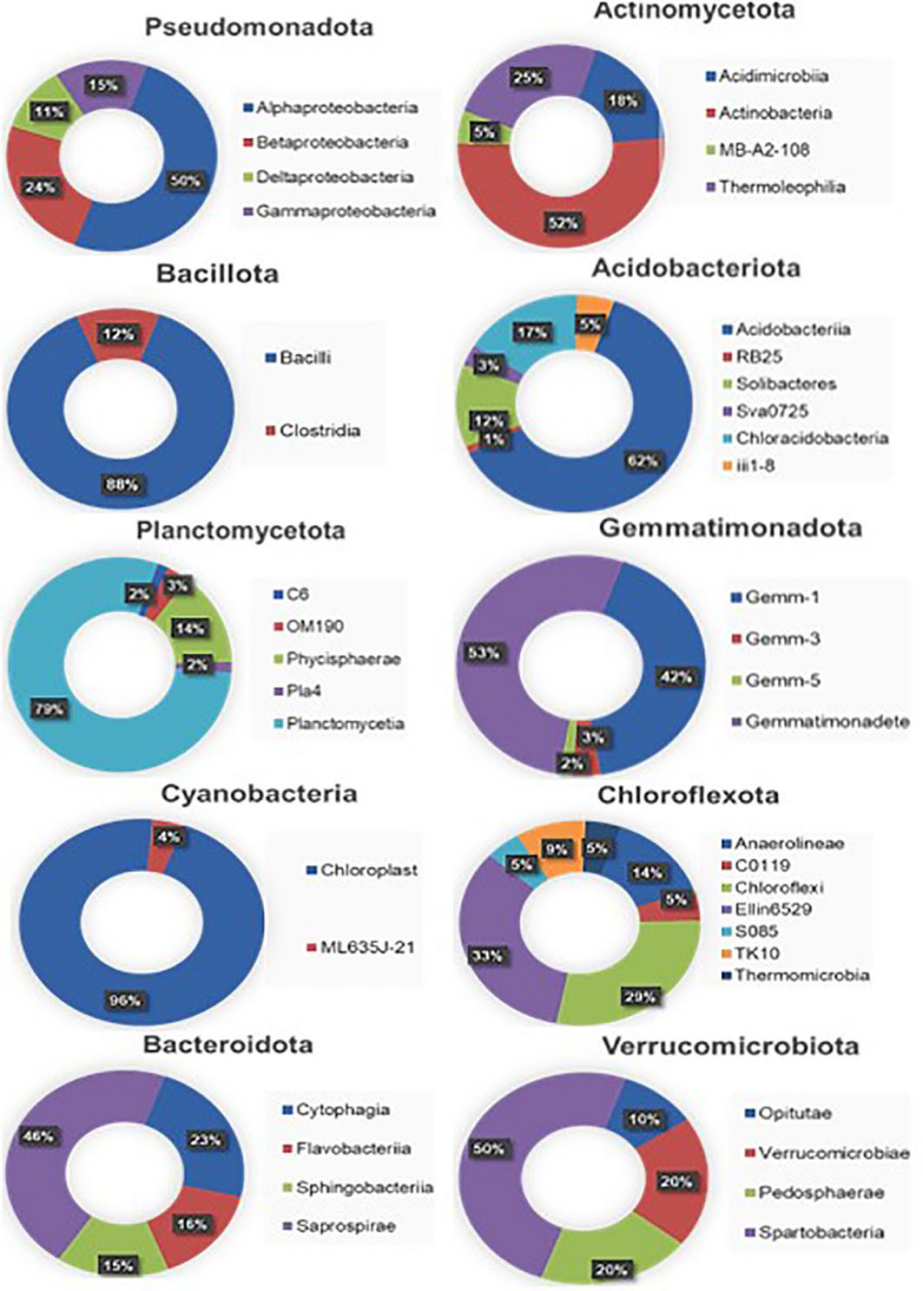
Taxonomic distribution of the soil metagenome at the class level. The decimal numbers are rounded off to their nearest values.

Among the Pseudomonadota, alphaproteobacteria (> 50%) were the dominant class followed by betaproteobacteria (> 24%), gammaproteobacteria (> 15%), and deltaproteobacteria (>11%) (**Figure 2**). In the case of Actinomycetota, the Actinobacteria (> 52%) class had the highest dominance followed by Thermoleophilia (> 25%). Similarly, in Bacillota and Planctomycetota, Bacilli (> 88%) and Panctomycetia (> 79%) have the highest dominance, respectively. In Acidobacteriota, 6 classes were identified, among which Acidobacteriia (> 62%) had the highest abundance. Furthermore, among Gemmatimonadota, Bactteroidota, and Verrucomicrobiota, Gemmatimonadetes (> 53%), Saprospirae (> 46%), and Spartobacteria (> 50%), respectively were found dominant. Phylum Chloroflexota showed the existence of a maximum of 7 classes with the dominance of candidate class Ellin6529 (> 33%) followed by Chloroflexi (>29%).

**Figure 3:**
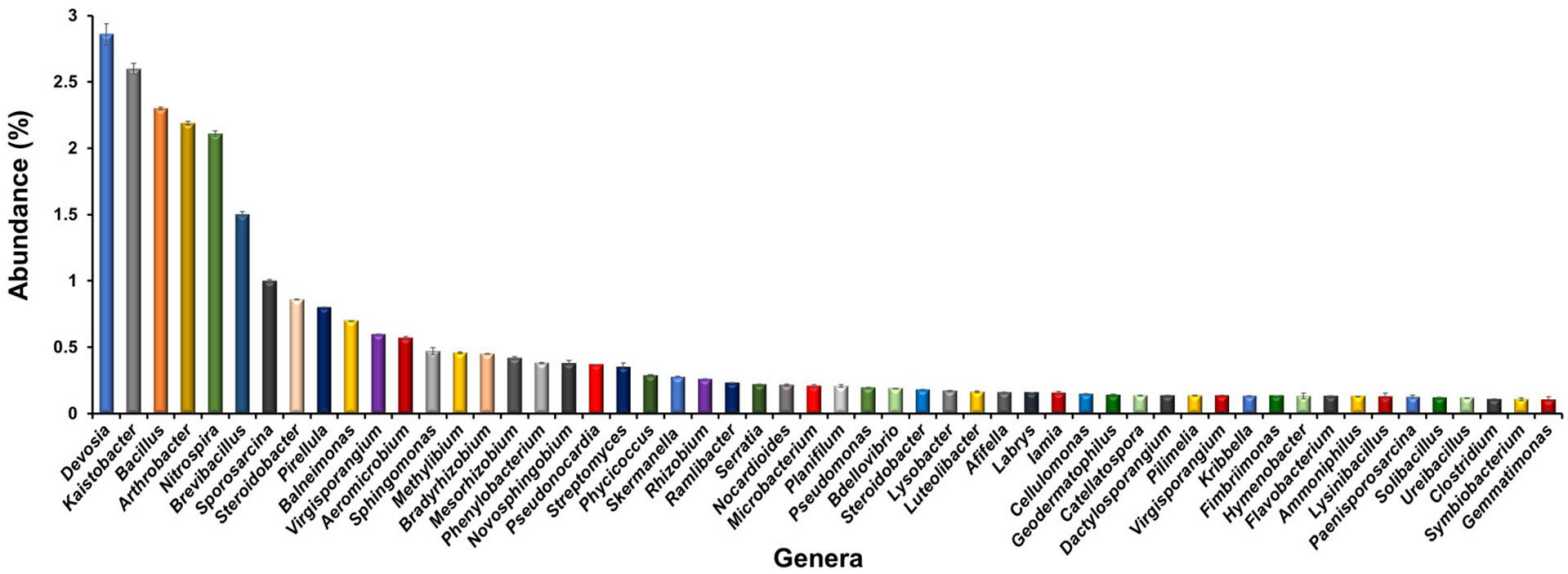
Bacterial genera observed in the rhizosphere of *L. camara* L. Genera with ≥0.1% abundance have been listed only.

A deeper insight up to the genus level revealed the existence of 54 different genera with more than ≥0.1% abundance in the rhizosphere of *L. camara* L. (**Figure 3**). *Devosia* was the dominant genus with 2.8% abundance followed by *Kaistobacter* (2.6%), *Bacillus* (2.3%), *Arthrobacter* (2.3%), *Nitrospira* (2.3%), *Brevibacillus* (1.5%) and *Sporosarcina* (1.0%). The other important genera observed were *Steroidobacter* (0.9%), *Pirellula* (0.8%), *Sphingomonas* (0.47%)*, Methylibium* (0.46%)*, Bradyrhizobium* (0.45%)*, Mesorhizobium* (0.42%), *Phenylobacterium* (0.38%) *Streptomyces* (0.35%)*, Rhizobium* (0.26%), *Microbacterium* (0.21%) *Serratia* (0.22%) *Pseudomonas* (0.2%), *Bdellovibrio* (0.19%) *Lysobacter* (0.17%) *Lamia* (0.16%), *Cellulomonas* (0.15%)*, Microbacterium* (0.21%)*, Flavobacterium* (0.13%), *Ammoniphilus* (0.13%), *Clostridium* (0.11%), *Gemmatimonas* (0.11%).

The diversity indices revealed the Simpson and Shannon values with 5.28 x 10^-3^ ± 6.31 x 10^-4^ and 8.22 ± 0.15, respectively. Among species richness estimates, Chao index had shown the value of 3.61 x 10^3^ ± 1.59 x 10^2^ (**Table 2**). Similarly, Ace index had a value of 4.92 x 10^3^ ± 2.11 x 10^2^.

**Table 2:** Diversity indices observed in the rhizosphere metagenome of *L. camara* L. Each value is the mean of three replicates.

OTUs	30024
Simpson	5.28 x 10^-3^ ± 6.31 x 10^-4^
Shannon	8.22 ± 0.15
Chao	3.61 x 10^3^ ± 1.59 x 10^2^
ACE	4.92 x 10^3^ ± 2.11 x 10^2^

Values in bracket indicate standard error.

### 3.3 Functional characterization of soil metagenomes

**Figure 4:**
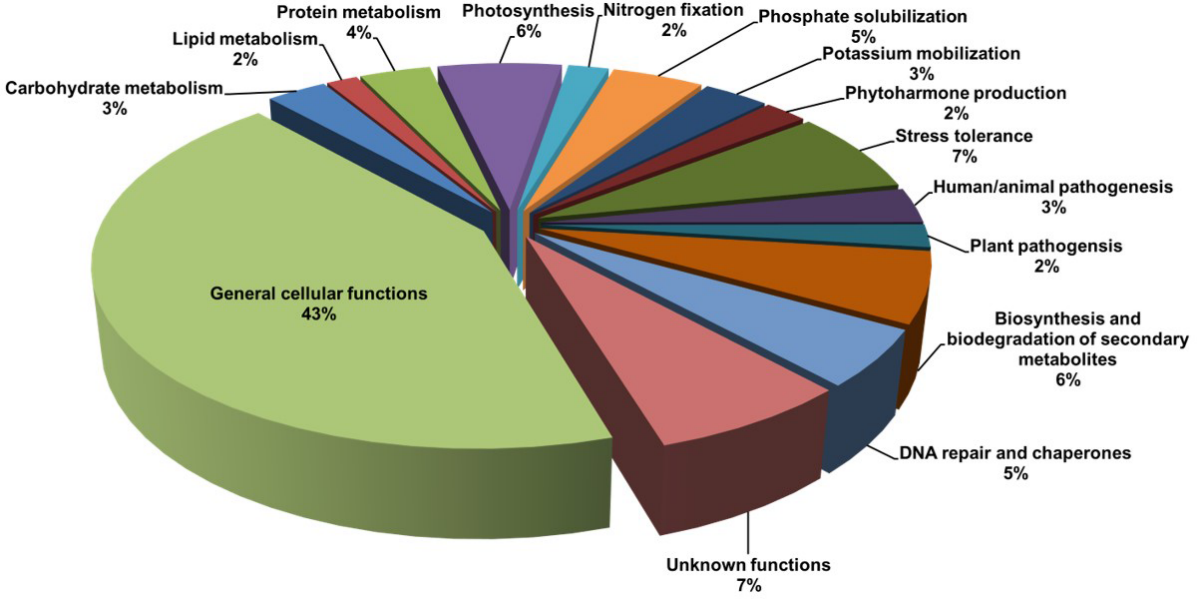
Functional characterization of the rhizosphere metagenome of *L. camara* L., predicted through PICRUSt analysis.

PICRUSt analysis predicted various functional traits in the soil metagenome (**Figure 4**). The majority of the OTUs were found associated with General cellular functions (> 43%) followed by stress tolerance (>7%). The other dominant functions were photosynthesis (> 6%), biosynthesis and biodegradation of secondary metabolites (> 6%), and DNA repair (> 5%). The analysis also predicted the functions associated with plant growth promotion, viz., phosphate solubilization (>5%), potassium mobilization (3%), and nitrogen fixation (2%) in the rhizosphere of *L. camara* L. A good proportion of the OTUs were found associated with unknown functions (> 7%).

## 4 Discussion

*L. camara* L. grows and flourishes under nutrient-limiting conditions, adverse climates, diverse habitats, and on a variety of soil types without any input or care. Previous studies have related this potential of the weed plants with the associated microbiome (Marasco et al., 2012; Panke-Buisse et al., 2015). It has been observed that *L. camara* L. invasion improves nitrogen, phosphorus, and enzyme activities in the soil (Fan et al., 2010; Weidenhamer and Callaway, 2010). It can be related with the presence of a diverse and potential rhizosphere microbiome of this plant (Kapoor and Kanwar, 2019; Proborini and Yusup, 2021). Similar observations have also been reported from other weeds (Kregiel et al., 2018). Interestingly, Sturz et al. (2001) have revealed a higher abundance of PGPR in a weed rhizosphere than in crop plants collected from the same agricultural field. Therefore, weed plants must be targeted to identify the diversity and community structure of associated microbiomes.

Illumina-based next-generation sequencing technique was employed to analyze the rhizosphere metagenome of the *L. camara* L. To the best of our knowledge, this study for the first time documented the *L. camara* L. microbiome. The study revealed that the majority of the OTUs belonged to bacteria and very few were from archaea. Rodriguez-Caballero et al. (2017) have analyzed the rhizosphere microbiome of an invasive weed *Pennisetum setaceum* through the pyrosequencing technique. They observed 22 phyla among which seven had shown ≥1% abundance with Pseudomonadota as the dominant phylum (38%) followed by Actinomycetota (25%). These results are in good accordance with our study in which Pseudomonadota and Actinomycetota were the most abundant phyla. Furthermore, Actinomycetota is known to possess the good decomposing activity and stress-tolerant ability and thus, they are likely to contribute to *L. camara* L. survival under adverse conditions (Suyal et al., 2021). In another study, Wang et al. (2022) analyzed the rhizosphere microbiome of invasive grasses *Phragmites*. Similar to our study, they reported the dominance of the phyla Pseudomonadota, Bacillota Actinomycetota, and Bacteroidota. Moreover, they also reported archaeal phyla Thermoproteota and Euryarchaeota. Rhizosphere archaea play an important role including ammonia oxidation, methanogenesis, abiotic stress tolerance, and plant growth promotion (Wang et al., 2022). Unfortunately, the comparison and establishment of any correlation between the weed plants and the associated rhizosphere microbiome could not be accomplished, due to the availability of scarce information on this subject.

At the genus level, Rodriguez-Caballero et al. (2017) observed the highest abundance of *Skermanella* and *Nocardioides*. Contrary to these results, the most abundant genera in our study were *Devosia*. Although, *Skermanella* and *Nocardioides* were also reported with lesser abundances. *Devosia, Kaistobacter*, and *Skermanella* are generally well known for heavy metal accumulation and bioremediation of polluted sites (Tian-Peng et al., 2021; Garbini et al., 2022). They are also reported to provide abiotic stress tolerance and plant growth promotion (Lopes et al., 2021). In the present study, isolation site of rhizosphere soil samples was located at hilly barren dry land having bushy plants. The temperature of this region is also very fluctuating, ranging from 37°C in summers to 0°C at winters. Even diurnal temperature also varies significantly. Therefore, presence of *Devosia, Kaistobacter*, and *Skermanella* seems very important in survivability of *L. camara* L. under such conditions. However, culture dependent studies and stress tolerant assays are required to unravel their exact role in this scenario.

The PICRUSt analysis predicted a good proportion of the functions belonged to plant growth promotion and stress tolerance. It can be well related with the dominance of agriculturally important genera in the rhizosphere *viz*. *Devosia, Kaistobacter Bradyrhizobium, Rhizobium, Microbacterium, Pseudomonas, Bacillus* and *Arthrobacter*. Among these, *Rhizobium* and *Bradyrhizobium* are established symbiotic nitrogen fixers with legumes. *Pseudomonas, Bacillus* and *Arthrobacter* have been applied to various crops and showed significant increase in crop productivity and abiotic stress tolerance ability of the plant (Joshi et al., 2019; Sahu et al., 2021; Suyal et al., 2021). Recently, Fu et al. (2020) and Paravar et al. (2023) have identified plant growth promoting *Pseudomonas* and *Kaistobacter* that enhanced the growth of *Lolium perenne* and *Ophiopogon japonicas*.

Very few reports are available which evaluated the PGPR potential of weed associated microorganisms under *in-situ* conditions. Zia et al. (2021) isolated three PGPR strains of genus *Proteus, Pseudomonas* and *Cronobacter* from the rhizosphere of desert weeds and employed them for improving drought resistance in the wheat. They observed that besides improving 20% more drought tolerance they produced plant growth hormone and siderophores. Their inoculation significantly enhanced plant growth and biomass under water-stress conditions. Therefore, identified genera in this study, especially *Devosia, Bacillus*, and *Pseudomonas*, need to be targeted for the growth of native crops in the future.

It is well-known fact that only a minor fraction of the microbiome can be cultivated in labs. It is generally due to the complex interactions of the microorganisms existing in the soil and their specific nutritional requirement that can never be mimicked under *in-vitro* conditions. Furthermore, in the present study, 5.6% of the OTUs do not belong to the existing taxa and thus, remained unclassified. They may be novel taxa that are still unidentified. Therefore, such uncultivated and unclassified rhizosphere microorganisms must be targeted in the future for utilizing their full potential in achieving sustainable agricultural goals.

## 5 Conclusion

The majority of the Indian weed plants have not gained much attention in microbiology-related studies. From this perspective, this study is useful in minimizing this knowledge gap to some extent. The findings revealed the vast diversity of prokaryotic communities in the *L. camara* L. rhizosphere with the existence of 41 bacterial phyla and 54 different genera. Among these, *Devosia* was the most dominant genera and need detailed characterization through culture-dependent studies. Further, PICRUSt analysis predicted the important role of bacterial diversity in stress response and plant growth promotion. However, more temporal and spatial analyses will be required to get a clearer picture of it.

## Data availability statement

The datasets presented in this study can be found in online repositories. The names of the repository/repositories and accession number(s) can be found below: https://www.ncbi.nlm.nih.gov/, SAMN29618131.

## Author contributions

UG, SK, TK: Conducted the experiments, and manuscript preparation. KP, NB, JA: Editing, review, and finalization of the manuscript. HD, DM, MS: Data analysis, and manuscript preparation. DS: Conceptualization of the work, data analysis, editing, and finalization of the manuscript. All authors contributed to the article and approved the submitted version.
