# Supplementary material for: Prokaryotic diversity and community structure in the rhizosphere of Lantana weed (Lantana camara L.)
Source: Front Plant Sci. 2023 Apr 21;14:1174859. doi: 10.3389/fpls.2023.1174859 (PMC10160613; doi:10.3389/fpls.2023.1174859)
Supplement: Supplementary file 1 [file Table_1.docx]

**SUPPLEMENTARY MATERIALS**

**Soil Analysis**

**1. Oxidizable organic carbon**

Oxidizable organic carbon of rhizosphere soils was measured by using the method given by Walkley and Black (1934). The details are given below:

**Reagents**

- H_3_PO_4_, 85%.
- H_2_SO_4_, concentrated (96%).
- NaF, solid.
- Standard 0.167M K_2_Cr_2_O_7_: Dissolve 49.04 g of dried (105°C) K_2_Cr_2_O_7_ in water & dilute to 1 L.
- 0.5 M Fe^2+^ solution: Dissolve 196.1 g of Fe(NH_4_)_2_(SO_4_)•6H_2_O in 800 mL of water containing 20 mL of concentrated H_2_SO_4_ and dilute to 1 L. The Fe^2+^ in this solution oxidizes slowly on exposure to air so it must be standardized against the dichromate daily.
- Ferroin indicator: Slowly dissolve 3.71 g of o-phenanthroline and 1.74 g of FeSO_4_•7H_2_O in 250 mL of water

**Procedure:**

- Weigh out 0.10 to 2.00 g dried soil (ground to < 60 mesh) and transfer to a 500-mL Erlenmeyer flask. The sample should contain 10 to 25 mg of organic C (17 to 43 mg organic matter). For a 1 g soil sample, this would be 1.2 to 4.3% organic matter. Use up to 2.0 g of sample for light colored soils and 0.1 g for organic soils.
- Add 10 mL of 0.167 M K2Cr2O7 by means of a pipette.
- Add 20 mL of concentrated H2SO4 by means of dispenser and swirl gently to mix. Avoid excessive swirling that would result in organic particles adhering to the sides of the flask out of the solution.
- Allow to stand 30 minutes. The flasks should be placed on an insulation pad during this time to avoid rapid heat loss.
- Dilute the suspension with about 200 mL of water to provide a clearer suspension for viewing the endpoint.
- Add 10 mL of 85% H3PO4, using a suitable dispenser, and 0.2 g of NaF. The H3PO4 and NaF are added to complex Fe3+ which would interfere with the titration endpoint.
- Add 10 drops of ferroin indicator. The indicator should be added just prior to titration to avoid deactivation by adsorption onto clay surfaces.
- Titrate with 0.5 M Fe^2+^ to a burgundy endpoint. The color of the solution at t2he beginning is yellow-orange to dark green, depending on the amount of unreacted Cr_2_O_7_^2-^ remaining, which shifts to a turbid gray before the endpoint and then changes sharply to a wine red at the endpoint. Use of a magnetic stirrer with an incandescent light makes the endpoint easier to see in the turbid system (fluorescent lighting gives a different endpoint color). Alternatively use a Pt electrode to determine the endpoint after step 5 above. This will eliminate uncertainty in determining the endpoint by color change. If less than 5 mL of Fe2+ solution was required to backtitrate the excess Cr_2_O_7_^2-^ there was insufficient Cr_2_O_7_^2-^ present, and the analysis should be repeated either by using a smaller sample size or doubling the amount of K_2_Cr_2_O_7_ and H_2_SO_4_.
- 9. Run a reagent blank using the above procedure without soil. The blank is used to standardize the Fe^2+^ solution daily.

**Calculation:**

Oxidizable Organic C (%) = $\frac{(B-S) x M of Fe2+ x 12 x 100}{g of soil x 4000}$

where:

B = mL of Fe^2+^ solution used to titrate blank

S = mL of Fe^2+^ solution used to titrate sample

12/4000 = milliequivalent weight of C in g.

**2. Available nitrogen**

The available nitrogen in soil was estimated by alkaline permanganate procedure given by Subbiah and Asija (1956) and expressed in terms of kg ha^-1^. Under this the four replicates were pooled and analyzed.

**Reagents:**

- 0.32% KMnO4 solution: Dissolve 3.2 g KMnO4 in 250-300 ml distilled water in a 1 L volumetric flask, make up the volume and mix well.
- 2.5% NaOH solution: Dissolve 25 g NaOH flakes in 250-300 ml distilled water, mix well and make up the volume upto 1 L
- Liquid paraffin (extra pure)
- 0.02 N H_2_SO_4_
- Boric acid: indicator solution: dissolve 20 g boric acid (H_3_BO_3_) in 700 ml hot water. Transfer the cooled solution to 1 l volumetric flask containing 200 ml of ethanol and 20 ml of mixed indicator. Mix the contents of the flask and add a little 0.02N NaOH solution until the colour is pink. Make up the volume and mix the contents of the volumetric flask.
- Mixed indicator: Dissolve 0.07 g of methyl red and 0.1 g bromocresol green in 100 ml of 95% ethanol.

**Procedure:**

Five gram of soil was taken in 500 ml Kjeldahl flask. Twenty mililitres distilled water was added and the contents were mixed properly. Then, 1 ml liquid paraffin was added with few glass beads. Now, 25 ml each of KMnO_4_ and NaOH solution was added. This mixture was subjected to distillation in a kjeldahl assembly and the liberated ammonia was collected in a 250 ml conical flask containing 20 ml of boric acid solution. Nearly 100 ml of distillate is collected in about 20 min. Titrate the distillate with 0.02 N H_2_SO_4_ upto the end point (pink colour).

**Calculation:**

% Available N = $\frac{\left( A-B \right)\times\left( N of acid \right)\times0.014\times100}{weight of soil (g)}$

A= titrate value of soil samples (ml)

B= titrate value of blank (ml)

N= Normality of acids used for titration

Available N (kg ha^-1^) = $\frac{\% available N\times2240000}{100}$

**3. Available phosphorous**

Phosphorous content in soil was estimated via **Olsen’s** method **(1954)** followed by **Das *et al.* (2003).**

**Reagents**

- Sodium bicarbonate 0.5 M : 84 g NaHCO_3_ was dissolved in water and made upto 2 L. pH adjusted to 8.5 with 1 M NaOH (4 g NaOH/ 100 ml) solution.
- Reagent A: Reagent A: 12 g ammonium molybdate [(NH_4_)_6_Mo_7_O_24._4H_2_O] dissolved in 250 ml of dH_2_O. 0.2908 g antimony potassium tartarate [K(SbO)C_4_H_4_O_6_. ½ H_2_O] is dissolved in 100 ml dH_2_O. Both solutions were added to 1000 ml of 2.5 M H_2_SO_4,_ properly mixed and made upto 2000 ml. Stored in dark and cool place.
- Reagent B: 1.056g ascorbic acid (C_6_H_8_O_6_) dissolved in 200 ml reagent A and mixed. Kept not more than 24 hrs at room temperature.
- Sulphuric acid 2.5 M: 140 ml of conc. H_2_SO_4_ was diluted to 1 L.
- Standard stock P solution: Exactly 0.439 g potassium dihydrogen orthophosphate (KH_2_PO_4_) was dissolved in 500ml of dH_2_O. 25 ml of 7N H_2_SO_4_ was mixed and volume made up to 1 L with dH_2_O. This results in 100 ppm phosphorus stock solution. From this, 50 times dilution ends in 2 ppm solution.

**Standard curve**

Phosphorus solution amount 1, 2, 3, 4, 5 and 10 ml of 2 ppm were taken in 25 ml volumetric flasks (**Appendix Va**). Five ml of extracting solution (Olsen’s) was added to each. The solution was acidified with 2.5 M H_2_SO_4_ to pH 5.0 and vaolume was made upto 20 ml with dH_2_O and 4 ml of reagent B was added. Volume was made up with dH_2_O and mixed. Blank was prepared with NaHCO_3_ solution, dH_2_O and 4 ml reagent B. After 10 min, the intensity of the blue colour was estimated by spectrophotometer at 730 nm **(Watanable and Olsen, 1965).**

**Procedure**

2.5 g of air dried soil was taken in a 150 ml conical flask. One small piece of Darco G60 or equivalent grade phosphorus- free activated charcoal was added. Further, Olsen’s reagent (50 ml; soil:solution ratio - 1:20) was added and the flask was kept on the reciprocating shaker for 30 min (180 oscillations min^-1^). A blank was run without soil. After 30 min., the solution was filtered quickly through Whatman no. 40 filter paper into clean beaker. 5 ml aliquot of extract was taken in 25 ml volumetric flask and acidified with 2.5 M H_2_SO_4_ to pH 5.0. Further, 20 ml dH_2_O was added followed by 4 ml of reagent B. It was left to stand for 10 min and intensity of blue colour on a spectrophotometer at 730 nm.

**Calculation:**

Available P (kg/ha) = $\frac{R x Vol. of extract}{Vol.of aliquot}$ x$\frac{2.24 x 106}{Wt.(g) of soil x 106}$

Where R= μg P in aliquot (obtained from standard curve)

$\frac{Rx 50 x 2.24}{5 x 2.5}\times$ μg P x 8.96

**4. Available potassium**

Available potassium was extracted from soils using neutral N ammonium acetate at 1:5, soil to extractant ratio and the concentration of potassium in the extract was determined by Flame photometer **(Jackson 1973).**

**Reagents**

- **Ammonium acetate (NH4OAc), 1.0 M solution**: Add 57 ml of conc. acetic acid to 700-800 ml distilled water in a 1-litre beaker. Then, with constant stirring, add 68 ml of conc. ammonium hydroxide. Bring the volume close to 1 l, and adjust pH to 7.0 by the addition of more ammonium hydroxide or acetic acid. Adjust pH to 7.0.
- **Potassium chloride (KCl) 0.02 M in 1.0 M ammonium acetate**: Dissolve 1.491 g of potassium chloride in one litre of 1.0 M NH4OAc solution to be used for preparing standard curve.

**Standard curve**

Prepare a series of working K standard solutions in the range of 0-50 mg K l^-1^ were prepared in extraction solution **(Appendix Vb).**

**Procedure**

5 g soil was placed in 150 ml conical flask and 25 ml of extracting solution (1 N NH4OAc, pH=7.0) was added. The content was kept on shaker for 5 min at 180 oscillations per minute. The content was immediately filtered through ordinary filter and filtrate was collected in a beaker. 5 ml of filtrate was transferred in 25 ml of volumetric flask and volume was made up with distilled water. The concentration of K in the filtrate was measured by using flame photometer. The concentration of K was calculated with the help of standard curve.

**Calculation**

Available K (kg ha^-1^) = $\frac{C \times volume of extract \left( \mathrm{ml} \right)\times2.24}{weight of soil (g)}$

**4. Calculation of Colony-forming units (CFU)**

One gram of the soil sample was homogenized within 9 mL of autoclaved distilled water. Serial dilutions ranging from 10^-1^ to 10^-5^ were prepared and vortexed vigorously. Further, 500 μL of diluted samples were plated separately on a respective growth medium [“Nutrient agar medium” (for total bacterial count)/ “Burk’s medium (for nitrogen fixers)/ Pikovskaya’s agar (for P-solubilizers)] , and the plates were incubated at 30 °C for 24 h. Further, colony-forming units (CFU) in one gram of soil sample were counted with the following formula:

$$\mathrm{CFU}g^{-1}=\frac{No. of bacterial colonies x dilution factor}{Volume of culture plated}$$
